# Effectiveness of Rehabilitation for Knee Osteoarthritis Associated With Isolated Meniscus Injury: A Scoping Review

**DOI:** 10.7759/cureus.34544

**Published:** 2023-02-02

**Authors:** Masateru Hayashi, Shusaku Koga, Takashi Kitagawa

**Affiliations:** 1 Department of Rehabilitation, Hanamizuki Orthopaedics Sports Clinic, Kiyosu, JPN; 2 Department of Rehabilitation Center, Sanno Hospital, Minato, JPN; 3 Department of Physical Therapy, School of Health Sciences, Shinshu University, Matsumoto, JPN

**Keywords:** lower limb pain, adl (activities of daily living), quality of life, exercise therapy, rehabilitation program, physical therapy modalities, knee injuries, meniscus tear, knee osteoarthritis/ koa

## Abstract

Meniscus tear is the most common type of injury to the meniscus and occurs more frequently on the medial compartments than the lateral compartments. Further, it is often caused by trauma or degenerative processes and can occur anywhere on either the meniscus, anterior horn, posterior horn, or midbody. Treatment of meniscus injuries is likely to greatly impact the evolution of osteoarthritis (OA) as meniscus injuries can gradually progress to knee OA. Hence, treatment of these injuries is important for managing the progression of OA. While the types of meniscus injuries and symptoms have been reported previously, the effectiveness of rehabilitation according to the degree of meniscus injury (e.g., vertical, longitudinal, radial, and posterior horn tears) remains unknown. In this review, we aimed to investigate whether rehabilitation for knee OA associated with isolated meniscus injuries varies with the degree of injury and determine the effects of rehabilitation on outcomes. We searched PubMed, Cumulative Index to Nursing and Allied Health Literature, Web of Science, and Physiotherapy Evidence Database for studies published before September 2021. Studies on ≥40-year-old patients with knee OA and isolated meniscus injury were included for analysis. The types of meniscus injury were classified as longitudinal, radial, transverse, flap, combined, or avulsion of the anterior and posterior roots of the medial meniscus, and assigned knee arthropathy grades of 0-4 according to the Kellgren-Lawrence classification. The exclusion criteria were meniscus injury, combined meniscus and ligament injury, and knee OA associated with combined injury in patients <40 years of age. There were no restrictions on the region, race, or gender of participants, or language or research format of the studies. The outcome measures were the Knee Osteoarthritis Outcome Score, Western Ontario and McMaster Universities Osteoarthritis Index Score, Visual Analog Scale or Numeric Rating Scale, Western Ontario Meniscal Evaluation Tool, International Knee Documentation Committee Score, Lysholm Score, 36-Item Short-Form Health Survey, one-leg hop test, timed up and go test, and re-injury and muscle strength. A total of 16 reports met these criteria. In studies that did not classify or distinguish degrees of meniscus injury, the effects of rehabilitation were generally favorable in the medium-to-long term. In cases where the intervention was not sufficiently effective, patients were recommended either arthroscopic partial meniscectomy or total knee replacement. Studies on medial meniscus posterior root tear did not confirm the effectiveness of rehabilitation due to the short intervention period. Further, Knee Osteoarthritis Outcome Score cut-offs, clinically important differences in Western Ontario and McMaster Universities Osteoarthritis Index, and minimum important changes in patient-specific functional scales were reported. Of the 16 studies reported in this review, nine met the definition. This scoping review has a few limitations such as the effect of rehabilitation alone could not be examined, and the intervention effectiveness differed at short-term follow-up. In conclusion, there was a gap in evidence regarding the rehabilitation of knee OA after isolated meniscus injury due to differences in intervention duration and methods. In addition, on short-term follow-up, intervention effects varied across studies.

## Introduction and background

Meniscus injuries are reported to occur in approximately 60% of individuals aged >50 years without knee pain due to degenerative changes in the knee joint [1]. The prevalence of meniscus injuries is estimated to be 4%-14% for individuals aged <40 years and 19%-43% for those aged ≥40 years. Additionally, the prevalence of meniscus injuries is estimated to be 14% for the medial meniscus and 5% for the lateral meniscus [2]. The age-standardized prevalence rate of knee osteoarthritis (OA) from 1990 to 2019 was reported to be as high as 122.42% and was higher in women than men [3]. Meniscus tears are also associated with knee OA. Knee OA is characterized by a gradual worsening of pain and loss of function in the knee joint [4]. Among 54 million Americans who have arthritis (most of whom have OA), 43% reported that their daily activities were limited by their arthritis [5]. Knee OA eventually leads to chronic disability due to involvement of the lower limb joints, which results in reduced fitness, and, ultimately, increased risk of cardiometabolic complications [6] and early mortality [7].

Research has not yet indicated whether meniscus degeneration is a causative factor for knee OA. However, knee arthritis begins with deterioration of the meniscus involving degenerative lesions and progresses to OA of the knee due to loss of meniscus function. Moreover, OA may cause extrusion of the meniscus and degenerative lesions in the knee joint as well as accelerating structural progression [8]. Morphological deformities of the meniscus (extrusion) and meniscus incompleteness (tears) are strongly related to the incidence and progression of knee OA [9]. Treatment of meniscus injuries is likely to play an important role in managing the progression of OA as meniscus injuries can eventually lead to knee OA. Meniscus tears and knee OA are known to cause pain and other symptoms. Treatment involves either conservative or surgical intervention [10,11]. Treatment of knee OA associated with meniscus injuries is initially conservative. Surgical treatment is preferred as the next option if the conservative treatment proves to be ineffective [5]. Rehabilitation, a form of conservative therapy, reportedly reduces pain and restores physical function in patients with knee OA [12,13]. Rehabilitation for medial and lateral meniscus injuries has been reported to be effective in the short and long term [14-16]. However, the difference in the effects of rehabilitation according to the degree of damage to the medial and lateral menisci (e.g., vertical, longitudinal, radial, and posterior horn tears) remains unclear.

While some studies have reported on the types of meniscus injuries and symptoms, the effectiveness of rehabilitation according to the degree of meniscus injury remains unknown. Therefore, this scoping review was conducted with two objectives. The primary objective was to assess whether knee OA with and without isolated meniscus injuries exhibits different rehabilitation outcomes according to the extent of medial and lateral meniscus injuries. The secondary objective was to determine how short-term and long-term outcomes after treatment change over time based on the extent of meniscus injury.

## Review

Methods

Overview

This scoping review was conducted to identify research findings related to isolated meniscus injury and knee OA and was conducted according to The Joanna Briggs Institute scoping review methodology [17] and Preferred Reporting Items for Systematic Reviews and Meta-Analyses (PRISMA) Extension for Scoping Reviews (PRISMA-ScR) guidelines [18]. This scoping review defined participants, concepts, and context (PCC) as requirements. The requirement for ethical approval of this study was waived considering the lack of participant involvement in this review. The review protocol was submitted to the “protocols.io” database for publication (dx.doi.org/10.17504/protocols.io.6qpvrd5w3gmk/v2).

Eligibility Criteria: Participants, Concepts, and Context Criteria

Types of participants: Patients aged ≥40 years with knee OA with isolated unilateral or bilateral meniscus injuries were included irrespective of their sex [2]. The inclusion criteria were as follows: knee OA grades 0-4 as defined by the Kellgren-Lawrence (KL) classification and traumatic or degenerative isolated meniscus injuries; the types of meniscus injuries were longitudinal, radial, horizontal, flap, and compound tears, as well as avulsion of the medial meniscus anterior and posterior root tears. The exclusion criteria were as follows: meniscus injuries in individuals aged <40 years, combined meniscus and ligament injuries (anterior cruciate ligament, posterior cruciate ligament, medial collateral ligament, and lateral collateral ligament), or knee OA associated with combined injuries. Additionally, cases with locking and catching due to meniscus injury, cartilage loss associated with traumatic meniscus injuries, patellofemoral OA, and surgical treatment (meniscectomy and repair) or orthotic therapy (immobilization in the acute phase of meniscus injury) were also excluded.

Concept: The intervention methods were physical therapy (PT) (including physical medicine), exercise therapy (ET), resistance training, strength training, neuromuscular training, and aerobic exercise. In studies with control groups, the control groups were classified as groups receiving placebo, usual care (conventional rehabilitation), and pharmacological therapy. Outcomes were assumed to include any physical outcomes usually treated by therapeutic interventions, such as pain, physical function, body mass index, stiffness, activities of daily living (ADL), and quality of life (QOL). Specifically, the Knee Osteoarthritis Outcome Score (KOOS), Western Ontario and McMaster Universities Osteoarthritis Index (WOMAC) score, visual analog scale (VAS) or numeric rating scale (NRS), Western Ontario Meniscal Evaluation Tool, International Knee Documentation Committee (IKDC) score, Lysholm score, 36-Item Short-Form Health Survey (SF-36), one-leg hop (OLH) test, timed up and go test, intermittent and constant assessment of pain, re-injury, and muscle strength (e.g., peak torque and total work) were assessed.

Context: The context of the study was intentionally broadened; however, we examined management strategies that could realistically be offered to patients in different settings. Therefore, no restrictions were placed based on the country or region of origin, race, sex, or language.

Types of Sources

Different study designs were targeted to identify gaps in evidence. Specifically, we included interventional studies (cluster randomized controlled trials (RCTs) and RCTs), observational studies (cohort, cross-sectional, and longitudinal studies), and case reports. Systematic reviews, meta-analyses, and narrative reviews were excluded.

Search Strategy

A comprehensive electronic search for studies on meniscus injuries was conducted using PubMed, Cumulative Index to Nursing and Allied Health Literature (CINAHL), Web of Science, and Physiotherapy Evidence Database (PEDro) databases. Further, sources of gray literature were searched using Open Gray [19]. A complete search strategy for these five databases was developed using keywords from the titles and abstracts of the relevant articles (Table 1). To conduct a comprehensive search for meniscus injuries, the search strategy was developed without including keywords related to OA. For a comprehensive literature search, we selected studies published from the inception of each database to September 20, 2021.

**Table 1:** Search strategy.

Search strategy
PubMed search strategy
(menisci, tibial [mh] OR menisc* [tiab] OR meniscus [mh] OR meniscus [tiab] OR meniscal [tiab] OR “tibial meniscus injuries” [mh]) AND (“physical therapy modalities” [mh] OR “physical therapy” [tiab] OR physiotherapy [tiab] OR kinesiotherapy [tiab] OR rehabilitation [mh] OR rehabilitation [tiab] OR “resistance training” [mh] OR “resistance training” [tiab] OR “strength training” [tiab] OR “neuromuscular training” [tiab] OR “exercise therapy” [mh] OR “exercise therap*” [tiab] OR “exercise program*” [tiab] OR “exercise training” [tiab] OR “aerobic training” [tiab] OR “aerobic exercis*” [tiab] OR “training program*” [tiab] OR “resistive exercis*” [tiab] OR “resistive training” [tiab] OR “endurance training” [mh] OR “endurance training” [tiab] OR “endurance exercis*” [tiab] OR Instructio* [tiab])
CINAHL search strategy
(MH menisci, tibial OR TI menisc* OR AB menisc* OR MH meniscus OR TI meniscus OR AB meniscus OR TI meniscal OR AB meniscal OR MH “tibial meniscus injuries”) AND (MH “physical therapy modalities” OR TI “physical therapy” OR AB “physical therapy” OR TI physiotherapy OR AB physiotherapy OR TI kinesiotherapy OR AB kinesiotherapy OR MH rehabilitation OR TI rehabilitation OR AB rehabilitation OR MH “resistance training” OR TI “resistance training” OR AB “resistance training” OR TI “strength training” OR AB “strength training” OR TI “neuromuscular training” OR AB “neuromuscular training” OR MH “exercise therapy” OR TI “exercise therap*” OR AB “exercise therap*” OR TI “exercise program*” OR AB “exercise program*” OR TI “exercise training” OR AB “exercise training” OR TI “aerobic training” OR AB “aerobic training” OR TI “aerobic exercis*” OR AB “aerobic exercis*” OR TI “training program*” OR AB “training program*” OR TI “resistive exercis*” OR AB “resistive exercis*” OR TI “resistive training” OR AB “resistive training” OR MH “endurance training” OR TI “endurance training” OR AB “endurance training” OR TI “endurance exercis*” OR AB “endurance exercis*” OR TI Instructio* OR AB Instructio*)
Web of Science search strategy
(meniscus OR “tibial meniscus injuries”) AND (“physical therapy modalities” OR “physical therapy” OR physiotherapy OR kinesiotherapy OR rehabilitation OR “resistance training” OR “strength training” OR “neuromuscular training” OR “exercise therapy” OR “exercise program*” OR “exercise training” OR “aerobic training” OR “training program*” OR “resistive exercis*” OR “resistive training” OR “endurance training” OR “endurance exercis*” OR Instructio*)
Physiotherapy Evidence Database (PEDro) search strategy
Abstract & Title: menisc* Therapy: Fitness training OR Strength training Body Part: Lower leg OR knee
Open Gray search strategy
Meniscus

Selection Process

PubMed, CINAHL, Web of Science, and PEDro databases were searched. The results were matched against all identified citations. Matching results were uploaded to Rayyan (Qatar Computing Research Institute, Ar Rayyan, Qatar), and duplicate references were removed [20]. Screening was assessed against the inclusion criteria for titles and abstracts after pilot testing by two independent reviewers (MH and SK). Additionally, selected full-text articles were evaluated against comprehensive criteria by two independent reviewers (MH and SK). Any disagreements between the reviewers at any stage of the selection process were discussed and resolved by the authors (MH and SK). If an agreement could not be reached, a third reviewer (TK) was consulted to resolve the issue.

Data Extraction

Data were extracted from the selected papers using Microsoft Excel® for Microsoft 365. Data extraction was specific to PCC as well as to study design findings relevant to the purpose of this scoping review. Data extracted included the author’s name and year of publication, country where the study was conducted, study design, study population (age and sex), sample size, purpose of the study, type of meniscus injury (medial and lateral meniscus, medial meniscus anterior, and posterior root tear), knee OA (KL classification), type and duration of intervention (including follow-up duration of intervention), outcomes, adverse events, and study limitations [21]. Additionally, for studies that compared rehabilitation with surgical treatment, only rehabilitation was considered for data extraction. Data extraction was conducted by two reviewers (MH and SK), and disagreements were discussed and resolved between them. In case of further disagreement, a third reviewer (TK) was consulted to adjudicate on the issue. As necessary, the authors of the original papers were notified if the information required for peer review was missing or if additional data were requested.

Data Analysis and Integration of Results

The PRISMA flowchart was used to present the search results and process of incorporation in a graphical form [22]. Additionally, a table summarizing the study characteristics, intervention and follow-up periods, intervention effects, adverse events, and limitations is presented. An online tool was used to create the diagram (https://www.mapchart.net/).

Results

Characteristics of the Included Studies

**Figure 1:**
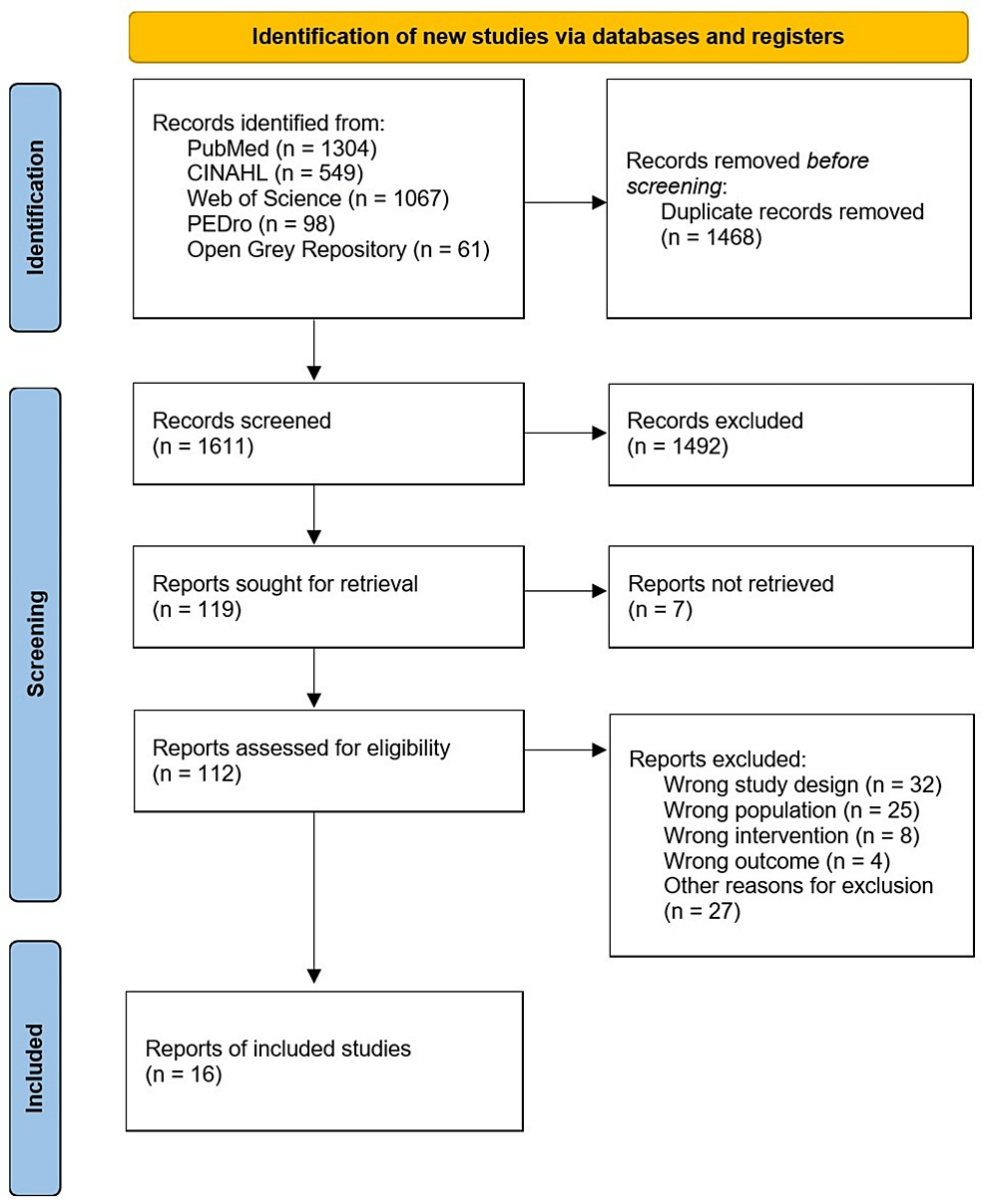
PRISMA flow diagram. PRISMA: Preferred Reporting Items for Systematic Reviews and Meta-Analyses

Overall, 3,079 titles and abstracts from the databases were matched to eliminate duplicate articles, leaving 1,611 papers. Primary screening of titles and abstracts was conducted based on the PCC eligibility criteria, and 112 studies were retained. Additionally, a secondary screening of 112 full-text articles based on the PCC eligibility criteria was conducted. Ultimately, 16 studies were included in this scoping review (Figure 1).

**Figure 2:**
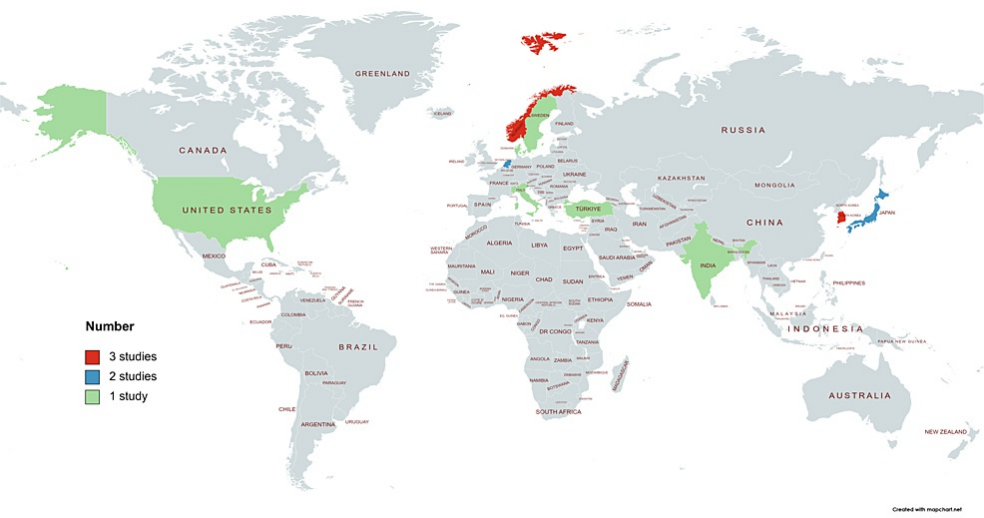
Distribution of studies by country.

The characteristics of the 16 studies that met the inclusion criteria are summarized in Figure 2 and Table 2.

**Table 2:** Characteristics of included studies. * ALL: sample size number, including patients who underwent surgical and conservative therapies. ** Conservative: non-surgical therapy (including exercise and drug therapy). *** SD: standard deviation **** Affected meniscus: medial, lateral, longitudinal vertical, horizontal, complex degenerative, radial, and vertical flap. ***** Meniscus ghost sign: medial meniscus posterior root radial tears. ****** Meniscus symptoms: clicking, catching, popping, giving way, pain with pivot or torque, pain that is episodic. BMI: body mass index; RCT: randomized controlled trial; KL: Kellgren-Lawrence classification; ET: exercise therapy; APM: arthroscopic partial meniscectomy; PT: physical therapy; MeTeOR: meniscal tear in osteoarthritis research; HAI: hyaluronic acid injection; OA: osteoarthritis; AAE: adapting alignment exercise; MTE: muscle training and exercise; MMPRT: medial meniscus posterior root tear

Author, year	Country	Study design	Sample size		Age^*** ^(years)	BMI^*** ^(kg/m^2^)	Types		Intervention	Comparison	Study purpose/Aims/Objectives
			ALL*	Conservative**			Meniscus injury	Knee osteoarthritis			
Graaf et al., 2018 [13]	Netherlands	Cluster RCT (crossover)	321	161	57.3 ± 6.8	27.2 ± 4.0	Affected meniscus****	KL: grades 0–3	ET	APM	To assess whether PT is non-inferior to APM for improving patient-reported knee function in patients with meniscal tears
Berg et al., 2020 [16]	Norway	RCT	140	58	50.3 ± 6.2	26.2 ± 4.0	Medial meniscus tear	KL: grades 0–2	ET	APM	To assess the course of radiographic features five years after ET or APM
Kise et al., 2019 [23]	Norway	Prospective RCT	107	55	50.1 ± 6.1	25.7 ± 4.0	Medial meniscal grades 2, 3a, and 3b	KL: grades 0–1	APM	ET	To identify the prognostic factors for two-year patient-reported outcomes in middle-aged patients with degenerative meniscal tears treated with ET or APM
Kise et al., 2016 [24]	Norway	Cluster RCT (crossover)	140	70	50.2 ± 6.2	26.4 ± 4.3	Meniscus degeneration	KL: grades 0–3	APM	ET	To determine whether ET is superior to APM for knee function in middle-aged patients with degenerative meniscal tears
Ahn et al., 2015 [25]	Korea	Retrospective study	41	13	62.3 ± 7.17	26.37 ± 4.01	MMPRT	KL: grades 1–4	MMPRT repair	PT	To compare the clinical results of conservative treatment and pull-out repair of MMRT and to analyze the prognostic factors of MMRT repair to determine repair indication
Lim et al., 2010 [26]	Korea	Retrospective study	30	30	59.0 (51–65)	Not defined	MMPRT	KL: grades 0–2	PT, medications	Not defined	To investigate the clinical results of non-operative treatment of degenerative (non-traumatic) posterior root tear of the medial meniscus
Yim et al., 2013 [27]	Korea	RCT	102	52	57.66 ± 11.0	26.46 ± 1.9	MMPRT (Horizontal tears)	KL: grades 0–1	PT, medications	APM	To compare the clinical results of arthroscopic meniscectomy and non-operative treatment for degenerative horizontal tears in MMPRT
Noorduyn et al., 2020 [28]	Netherlands	RCT	321	162	57.3 ± 6.8	27.2 ± 4.0	Meniscus tear	KL: grades 0–3	APM	ET	To compare APM with PT in patients with a degenerative meniscus tear, focusing on patients’ most important functional limitations as the outcomes
Ikuta et al., 2020 [29]	Japan	Prospective RCT	26	13	AAE: 67.9 ± 7.2 MTE: 68.2 ± 10.8	AAE: 22.9 ± 2.3 MTE: 24.2 ± 2.3	Meniscus ghost sign*****	KL: grades 1–2	AAE	MTE	To verify that exercise aimed toward improving knee kinematics reduces the knee adduction angle during walking and prevents rapid cartilage degeneration in the medial compartment of the knee
Kudo et al., 2013 [30]	Japan	RCT	209	81	Group: 63.8 ± 5.9 Home: 65.6 ± 5.8	Group: 23.8 ± 2.9 Home: 23.8 ± 3.0	Mink grades 0–3	KL: grades 0–4	ET (group)	ET (home)	To evaluate how mode of treatment delivery affects knee OA symptom improvement and to analyze potential factors affecting improvement after ET
Stensrud et al., 2015 [31]	Denmark	RCT	82	40	49.2 ± 6.4	26.9 ± 4.1	Medial meniscus tear	KL: grades 0–2	ET	APM	To compare the effects of a 12-week exercise therapy program and APM on knee strength and functional performance in middle-aged patients with degenerative meniscus tears
Neogi et al., 2013 [32]	India	Prospective study	33	33	55.8 (50–62)	Not defined	MMPRT	KL: grades 0–2	PT, medications	Not defined	To evaluate the effect of supervised ET on patients with MMPRT
Prati et al., 2017 [33]	Italy	Non-RCT (pilot study)	20	8	61.3 ± 4.3	Not reported	Meniscus tear	KL: grades 0–2	PT (MeTeOR)	APM	To compare the efficacy of PT and APM on physical function in patients with meniscus injuries and symptomatic knee OA
Sonesson et al., 2020 [34]	Sweden	RCT	146	61	54.0 ± 6.0	Not reported	Meniscus symptoms******	KL: grades 0–3	APM	PT	To evaluate whether arthroscopic knee surgery combined with an exercise program provides an additional five-year benefit over that of an exercise program alone in middle-aged patients with meniscal symptoms; to determine whether baseline mechanical symptoms affected outcomes; and to compare radiographic changes between treatment groups
Katz et al., 2013 [35]	United States	Cluster RCT (crossover study)	330	169	57.8 ± 6.8	30.0 ± 6.1	Meniscus symptoms******	KL: grades 0–3	APM	PT (MeTeOR)	To compare the effectiveness of APM with that of standard PT regimens
Başar et al., 2021 [36]	Turkey	Prospective RCT	146	45	50.9 ± 4.5	28.7 ± 2.2	Meniscus tear	KL: grades 1–3	APM, APM and HAI; PT and HAI	PT	To compare the effectiveness of APM and PT in degenerative meniscus tears and investigate the effect of HAI injection

Studies were conducted in various countries, primarily in Europe and Asia. The countries included Norway [16,23,24] and South Korea [25-27] with three studies each, the Netherlands [13,28] and Japan [29,30] with two studies each, and Denmark [31], India [32], Italy [33], Sweden [34], the United States [35], and Turkey [36], with one each. Eight studies did not classify or distinguish the degree of meniscus injury [13,24,28,30,33-36]. Additionally, there were eight studies on medial meniscus tears [16,23,25-27,29,31,32], and five examined medial meniscus posterior root tears (MMPRT) [25-27,29,32]. The studies that did not classify or distinguish the degree of meniscus injury included three cluster RCTs [13,24,35], four RCTs [28,30,34,36], and one non-RCT [33]. Moreover, studies on the medial meniscus included five RCTs [16,23,27,29,31], two of which were on MMPRT [27,29]. We were unable to find any studies on the lateral meniscus or anterior horn of the meniscus. Regarding comparisons of interventions for meniscus injury and knee OA, most studies included intervention and control groups. Specifically, among the studies that did not classify or distinguish the degree of meniscus damage, there were seven comparisons between APM and PT/ET [13,24,28,33-36], and one comparison between group and home exercises [30]. Moreover, regarding medial degenerative meniscus tears, there were three comparisons between APM and ET [16,23,30]. For MMPRT, one study compared APM and PT/medications [27], one compared MMPRT repair and PT [25], one evaluated PT and conventional ET [29], and two examined PT/medications only [26,32].

Meniscus Tear or Symptoms

The rehabilitation programs in the studies that did not classify or distinguish the degree of meniscus injuries included warming up, neuromuscular, and balance exercises around the knee and hip joints, as well as muscle strengthening exercises (Table 3).

**Table 3:** Details of treatment or intervention (rehabilitation program and medication). MeTeOR: meniscal tear in osteoarthritis research; e-stim: electrical stimulation; NMES: neuromuscular electrical stimulation; IFC: interferential current; LE: lower extremity; SAQ: short arc quadriceps; LAQ: long arc quadriceps; HS: hamstrings; ROM: range of motion; PM: physical medicine; ET: exercise therapy; PT: physical therapy; MTE: muscle training and exercise; AAE: adapting alignment exercise; NSAIDs: non-steroidal anti-inflammatory drugs

Author, year	Intervention duration	Sessions	Program		
			Warming up	Exercise/physical medicine	Cooling down
Graaf et al., 2018 [13]	8 weeks	16	Stationary bicycle	Calf raises on a leg press; standing hip extension in a “multi-hip” training device; balancing on wobble board with both feet; calf raises standing on one leg; leg presses with the shinbone placed horizontally and the knee starting at 110°; unilateral lunges with <90° knee flexion; balancing on wobble board with one foot with throwing a ball; cross-trainer for cardiovascular exercise; stair walking; walking; running; jumping (according to the patients’ icf with throwing a ball)	Stationary bicycle
Berg et al., 2020 [16]	12 weeks	24	Stationary bicycle	Neuromuscular exercises; squat; single-leg squat; step-up; knee stability in pull loop; hamstring on fitball; single-leg leg press; single-leg knee extension; single-leg leg curl; skating; limping cross	Not reported
Kise et al., 2019 [23]	12 weeks	24–36	Stationary bicycle	Neuromuscular exercises: squat; single-leg squat; step-up; knee stability in pull loop; hamstring on fitball; single-leg leg press; single-leg knee extension; single-leg leg curl; skating; limping cross	Not reported
Kise et al., 2016 [24]	12 weeks	24–36	Stationary bicycle	Squat; single-leg squat; step-up; knee stability in pull loop; hamstring on fitball; single-leg leg press; single-leg knee extension; single-leg leg curl; skating; limping cross	Stationary bicycle
Ahn et al., 2015 [25]	Not reported	Not reported	Not reported	PT: strengthening exercise	Not reported
Lim et al., 2010 [26]	Medication: 8–12 weeks	16	Not reported	Medication: NSAIDs	Not reported
	PT: ≥8 weeks			Stretching of knee extensors and flexors; range of movement in the hip, knee, and ankle in all directions; stationary bicycling; straight leg raises with one leg; concentric knee flexion with two legs; concentric knee extension with two legs; mini squat with <45° flexion with weight	
Yim et al., 2013 [27]	3 weeks	9	Not reported	Medication: NSAIDs; muscle relaxants	Not reported
				PT: muscle strength (knee extension in a sitting position, knee flexion in sitting position, half squats with <45° of flexion and weights, squats with full flexion and weights); endurance (stationary bicycling); flexibility (stretching of knee extensors and flexors)	
Noorduyn et al., 2020 [28]	8 weeks	16	Stationary bicycle	PT: calf raises on a leg press; standing hip extension in a “multi-hip” training device; balancing on wobble board on both feet; calf raises standing on one leg; leg presses with the shinbone placed horizontally and the knee starting at 110°; unilateral lunges with < 90° knee flexion; balancing on wobble board on one foot while throwing a ball; cross-trainer as cardiovascular exercise; stair walking; walking; running; jumping (according to the patients icf while throwing a ball	Stationary bicycle
Ikuta et al., 2020 [29]	Not reported	Not reported	Not reported	MTE: ROM exercise; muscle strengthening exercises; home exercises: ankle and knee ROM exercises; quadriceps strength; hip abduction strength; weight-bearing exercises	Not reported
				AAE: ROM exercises; muscle strengthening exercises; knee malalignment during the stance phase of gait (AAE, weight-bearing exercises); home exercise: ankle and knee ROM exercise; quadriceps strength; hip abduction strength	
Kudo et al., 2013 [30]	12 weeks	24	Stretching	ET (group): strengthening muscles around the knee, trunk, hip, and ankle using a combination of open leg kinetic chain exercises, isotonic and isometric squats and calf raises, stationary bicycle for 20–40 minutes at an exercise intensity of 55%–65% of predicted maximum heart rate; stabilization exercises on the stationary bicycle focusing on the knee joints and incorporating the pelvis and core; balance ball and balance cushion to improve standing postural balance and core muscle strength	Stretching
				ET (home): muscle strengthening; stabilization exercises	
Stensrud et al., 2015 [31]	12 weeks	24–36	Stationary bicycle	Neuromuscular exercises: squat; single-leg squat; step-up/down; knee stability in pull loop; one-leg flying-balance; skating; limping cross; lunges. Strength exercises: single-leg leg press; single-leg knee extension; single-leg leg curl; hamstring on fitball	Stationary bicycle
Neogi et al., 2013 [32]	12 weeks	30	Not reported	Medications: celecoxib 200 mg; ibuprofen sustained release 1,600 mg; NSAIDs; paracetamol 4 g/day; tramadol sustained release 100 mg	Not reported
				PT: stretching of knee extensors and flexors; range of movement in the hip, knee, and ankle in all directions; stationary bicycling; concentric knee flexion with two legs and eccentric knee flexion with one leg; knee flexion with one leg and gradually increasing resistance with a thera-band; straight leg raises with a weight attached to one leg (increased as tolerated); mini squat with <80° flexion without weights; mini squat with <80° flexion with weights	
Prati et al., 2017 [33]	5 weeks	10	Not reported	MeTeOR trial	Not reported
Sonesson et al., 2020 [34]	12 weeks	24	Stationary bicycle	PT: leg press; hip adductors; hip abductors; heel raise; ball squat; standing on a balance board on one leg	Not reported
				Home exercise: brisk walk; squat; pelvic lift; pelvic lift with a ball between the knees and one knee extended; heel raise; wall squat; standing on a pillow on one leg	
Katz et al., 2013 [35]	6 weeks	6–12	Not reported	MeTeOR trial (the three-stage structured program)	Not reported
				Phase I-acute phase: Decrease inflammation: retrograde massage; cryotherapy; electrical stimulation: NMES or IFC manual therapy: joint mobilization; soft tissue mobilization; stretching LE muscles; open chain exercises: quad sets SAQ/LAQ/HS curls hip-4-way closed chain exercises: bicycle; elliptical; treadmill; leg press; balance/proprioception	
				Phase II-subacute phase: Decrease inflammation: retrograde massage; cryotherapy; electrical stimulation: NMES or IFC manual therapy: joint mobilization; soft tissue mobilization; stretching LE muscles; open chain exercises: addition of more concentric/eccentric hip/knee progressive resistive exercises; ROM exercises; closed chain exercises: resisted terminal knee extension; modified mini squats; step up/down progressions; toe raises; functional and agility training	
				Phase III-advanced activity phase: Stretching program (continued); pre-therapeutic exercises program (continued); closed chain program with progression: dynamic single-leg stance; plyometrics; running; sport specificity training	
Başar et al., 2021 [36]	PM: 4 weeks	PM: 12	Not reported	Transcutaneous electrical nerve stimulation; low-intensity pulsed ultrasound	Not reported
	ET: 8 weeks	ET: 24		Neuromuscular and strength exercises (concentric and eccentric exercises in both weight-bearing and non-weight-bearing positions)	

The duration of exercise varied widely with each study, ranging from five to 12 weeks. The follow-up periods also varied considerably, from one month to five years (Table 4).

**Table 4:** Intervention results for rehabilitation. * Data brackets in outcome scores indicate 95% confidence intervals. ** The presentation of results in indicates the change from baseline, but if not mentioned in the original paper, it is presented as Baseline and Final F/U. *** KOOS scores at five years and change from baseline to five years for the full analysis set. **** KOOS Pain, function in ADL, and Tegner were not entered as no significant differences were reported. ADL: activities of daily living; QOL: quality of life; CID: clinically important difference; MIC: minimal important change; VAS: visual analog scale; F/U: follow-up; MD: mean difference; IKDC: International Knee Documentation Committee; KOOS4: knee osteoarthritis outcome score (pain, symptoms, function in sports and recreation, and quality of life); KOOS: knee osteoarthritis outcome score; WOMAC: Western Ontario and McMaster Universities Osteoarthritis Index; SF-36: 36-Item Short-Form Health Survey; ROM: range of motion; PSFS: patient-specific functional scale; EQ-VAS: EuroQol visual analog scale; Sport/Rec: sport/recreation; EQ-5D: EuroQol 5 dimensions; NRS: numeric rating scale; AROM: active range of motion; OLH: one-leg hop for distance; 6MTH: 6-meter timed hop; AAE: adapting alignment exercise; MTE: muscle training and exercise; TAS: Tegner activity scale

Author, year	Intervention duration	Follow-up duration	Change from baseline*; final follow-up data are indicated**			Cut-offs/CID/MIC
			Pain/symptoms	Physical function	ADL/QOL	
Graaf et al., 2018 [13]	8 weeks	3, 6, 12, and 24 months	VAS during weight-bearing; baseline: 59.3 mm, final F/U: 25.5 mm (MD, 32.5 mm [26.7–38.3])	IKDC knee function score; baseline: 46.5 points, final F/U: 67.7 points (MD, 20.4 points [17.5–23.2])	Not reported	Not reported
Berg et al., 2020 [16]	12 weeks	3 and 12 months, 2 and 5 years	KOOS***; Pain: 21.3 points (17.4–25.2), symptoms: 13.2 points (9.3–17.2)	Not reported	KOOS***; ADL, 14.6 points (11.5–17.8); Sport/Rec, 29.0 points (22.9–35.1); QOL, 30.7 points (25.5–36.0);	Not reported
Kise et al., 2019 [23]	12 weeks	2 years	KOOS; MD symptoms: a 1-s better hop test result was associated with better KOOS symptoms at 2.6 points (0.2–4.9 points)	Not reported	KOOS; MD Sport/Rec: a 1-s better hop test result was associated with better KOOS Sport/Rec score of 5.5 points (2.1–9.0 points), and QOL score of 4.2 points (0.7–7.7 points)	Not reported
Kise et al., 2016 [24]	12 weeks	3, 12, and 24 months	Not reported	Not reported	KOOS_4_; 25.3 points (21.6–29.0)	KOOS cut-offs; total: 10.1 points, pain: 7.4 points, symptoms: 8.4 points, function in sport and recreation: 10.9 points, ADL: 4.1 points, QOL: 13.6 points
Ahn et al., 2015 [25]	Not reported	18.40 ± 4.64 months	Not reported	Not reported	IKDC subjective score: 44.7±12.8 to 45.9±14.0 (P=0.633); Tegner and Lysholm activity scale: 51.6±23.1 to 51.2±22.7 (P=0.932)	Not reported
Lim et al., 2010 [26]	8–12 weeks	6 and 12 months, final F/U at 36 months (24–51 months)	VAS; Baseline: 71 ± 15 mm, final F/U: 31 ± 13 mm	Lysholm knee score; baseline: 67.0 (40.0–78.0), final F/U: 80.0 (72.0–96.0)	Not reported	Not reported
Yim et al., 2013 [27]	8 weeks	3 and 6 months, 1 and 2 years	VAS; Baseline: 49 mm, final F/U: 17 mm	Lysholm knee score; Baseline: 65.2, final F/U, 84.3	TAS score; Baseline: 4.1 (0.0–6.0), final F/U: 4.9 (0.0–8.0)	Not reported
Noorduyn et al., 2020 [28]	8 weeks	3, 6, 12, and 24 months	Not reported	PSFS score; MD 4.0 ± 3.1 points (6.7 ± 2.0 to 2.7 ± 2.5)	Not reported	MIC of PSFS: 2.5 points
Ikuta et al., 2020 [29]	6 months	6 months	VAS; AAE: -27.0 mm (-42.0–-11.9; p = 0.004) KOOS****; symptoms: MTE 15.7 points (6.4–24.9; p = 0.001)	Not reported	KOOS****; Sport/Rec: MTE, 16.5 (5.1–27.9; p = 0.001); AAE 26.2 (16.9–35.4; p = 0.005) KOOS****; QOL AAE, 19.2 (9.5–29.0; p < 0.009)	Not reported
Kudo et al., 2013 [30]	12 weeks	3 months	Not reported	Flexion contracture; group: ≥6 points 19 (54.3) %, ≤6 points 20 (43.5) %, home: ≥6 points 25 (30.9) %, ≤6 points 17 (41.5) % Quadriceps strength; group: ≥6 points 1.53 ± 0.51 Nm/kg, ≤6 points 1.29 ± 0.49 Nm/kg, home: ≥6 points 1.38 ± 0.52 Nm/kg, ≤6 points 1.38 ± 0.48 Nm/kg	Not reported	Not reported
Stensrud et al., 2015 [31]	12 weeks	3 months	Not reported	Muscle strength; isokinetic knee extension peak torque: 25.2 Nm (17.8–32.6; p < 0.05), isokinetic knee extension total work: 76.4 J (40.4–112.4), Isokinetic knee flexion peak torque: 12.7 Nm (7.7–17.8), isokinetic knee flexion total work: 64.4 J (25.2–103.5) OLH: 7.9 (3.2–12.6), 6MTH: 0.4 (0.1–0.6), knee-bending at 30 seconds 11.2 (8.5–13.8)	Not reported	Not reported
Neogi et al., 2013 [32]	12 weeks	3, 6, and 12 months	VAS: rest: baseline, 20 mm (0–30); final: F/U, 0 mm (0–40) (P=0.03); activity: baseline, 50 mm (20–70); final F/U, 10 mm (0–70) (P=0.04).	Lysholm knee score; baseline, 56 ± 8 (32–73); final F/U, 79 ± 7 (40–91) (p = 0.0212). TAS score: baseline, 2 (0–3); final F/U 4 (1–5) (p = 0.03)	Not reported	Not reported
Prati et al., 2017 [33]	5 weeks	1 and 3 months	NRS: rest: baseline, 5.1–0.1 (1 month); activity: baseline, 5.1–2.4 (1 month); KOOS; pain: 33.3 ± 17.4 points, symptoms: 25.9 ± 21.3 points	AROM; flexion: baseline, 123–134 (1 month); extension: baseline, -0.6–0 (1 month)	KOOS; ADL 28.5 ± 20.4 points, QOL 16.4 ± 21.1 points	Not reported
Sonesson et al., 2020 [34]	12 weeks	1, 3, and 5 years	KOOS; pain: baseline, 60.2 (55.4–65.1); final F/U, 86.0 (79.7–92.2); symptoms: baseline, 62.7 (57.6–67.9); final F/U, 85.6 (80.0–91.3); EQ-VAS: baseline, 65.9 (60.2–71.5); final F/U, 83.5 (79.0–88.0)	Not reported	KOOS; ADL: baseline, 70.2 (65.3–75.1); final F/U 87.9 (82.3–93.4) Sport/Rec: baseline, 36.9 (30.3–43.6); final F/U 65.9 (55.6–76.2) QOL: baseline, 37.5 (33.0–42.0); final F/U 68.1 (59.4–76.7) EQ-5D: baseline, 0.65 (0.59–0.71); final F/U 0.86 (0.81–0.91)	Not reported
Katz et al., 2013 [35]	6 weeks	3, 6, and 12 months	KOOS pain MD, 6 months: 21.3 points (18.4–24.2); final F/U: 27.3 points (24.1–30.4)	WOMAC physical-function score MD; 6 months: 18.5 points (15.6–21.5), final F/U: 22.8 points (19.8–25.8)	SF-36 physical-activity score MD; 6 months: 23.1 (19.2–27.0), final F/U: 28.1 (24.0–32.1)	WOMAC score CID; physical-function scale: 8 points
Başar et al., 2021 [36]	8 weeks	2 and 6 months	VAS; baseline: 69 ± 7 mm, final F/U: 20 ± 11 mm (p < 0.0001)	ROM; baseline: 102.3 ± 6.3, final F/U: 115.6 ± 6.1 (p < 0.0001)	Not reported	Not reported

Outcomes evaluated for the effects of exercise were pain and symptoms (VAS or NRS, EuroQol VAS [EQ-VAS], and KOOS pain subscale), physical function ([IKDC score, WOMAC physical function score, range of motion [ROM], and patient-specific functional rating scale [PSFS]), and ADL or QOL (KOOS subscales [sports/recreation, ADL, and QOL] and SF-36 physical activity score). Interventions tended to improve short-, medium-, and long-term pain and symptoms, physical function, and ADLs or QOL. In contrast, studies with a crossover design reported a shift to APM or total knee replacement (TKR) after rehabilitation (Table 5) [13,24,35].

**Table 5:** Summary of results, adverse events, and limitations of the included studies. APM: arthroscopic partial meniscectomy; MD: mean difference; TKR: total knee replacement; MCID: minimal clinically important difference; PT: physical therapy; ET: exercise therapy; MRI: magnetic resonance imaging; GRC: global rating of change scale; KOOS4: knee osteoarthritis outcome score (pain, symptoms, function in sports and recreation, and quality of life); IKDC: International Knee Documentation Committee; PSFS: patient-specific functional scale; OA: osteoarthritis; LSI: Limb Symmetry Index; NSAIDs: non-steroidal anti-inflammatory drugs

Author, year	APM/TKR crossover	Adverse events	Study limitations
Graaf et al., 2018 [13]	As-treated analysis: delayed APM (N = 47). IKDC score: delayed APM, from 40.8 points at baseline to 63.0 points at 24 months (MD, 21.5 points [95% CI, 15.8–27.3]). Knee pain during weight-bearing: delayed APM, from 66.4 mm at baseline to 36.0 mm at 24 months (MD, 27.5 mm [95% CI, 16.0–39.1])	Adverse events (e.g., cardiovascular, neurological, or internal medicine conditions, venous thromboembolism, or repeat knee surgery) N = 8. Non-serious adverse events (e.g., reactive arthritis, joint paint resulting in extra consultation or surgical site infection) N = 4	Screening logs for patients who were not randomized were not maintained. Non-inferiority margin based on 8.8 points, the smallest detectable change, is a conservative estimate of potentially relevant differences. Grouping was not blinded. Non-inferiority testing was intended for the secondary analyses, but no non-inferiority margins were specified in the protocol. MCIDs for the secondary outcomes were not defined until after data analyses. Radiographs were interpreted by a single radiologist. The combination of APM and PT may be more effective than APM alone
Berg et al., 2020 [16]	Not reported	Not reported	The radiographic clinics were instructed to follow a standardized protocol; however, we identified some deviations. No radiographic evaluations of the patellofemoral joint were performed. The study did not have sufficient power to detect differences in individual radiographic features. In non-surgical and surgical treatments, one-way crossover is a potential challenge; patients can cross over from ET to APM, but not from APM to ET once they have had surgery
Kise et al., 2019 [23]	Not reported	Not reported	This study did not include radiographs appropriate for evaluating varus and valgus alignment. MRI evaluation included degeneration grades 0–3b (lower is better) and measurement of meniscus extrusion. In the subgroup analyses of GRC scale pain and function, small sample sizes, especially for the APM group, might have led to spurious results; this is reflected in the wide 95% CIs. Possible low external validity
Kise et al., 2016 [24]	Crossover (1 patients with multiple surgeries) was 12 patients A comparison of KOOS_4 _at 12 months to 2 years between the crossover and ET groups showed no between-group difference (25.5 vs 25.5, p = 1.0)	23% of subjects experienced severe pain, swelling, instability, stiffness, and reduced range of motion	The lack of a sham surgery group; we cannot exclude the possibility that the greater placebo effect from surgery on patient-reported outcomes masks a “real” difference in treatment between groups
Ahn et al., 2015 [25]	Not reported	Not reported	The follow-up period was short and the cohort size was small. There were significant differences in preoperative demographics and clinical characteristics such as age. Mental health is a component of patient satisfaction, but was not assessed
Lim et al., 2010 [26]	Not reported	Not reported	Non-operative treatment was not compared with surgical treatments such as arthroscopic meniscectomy, repair, or osteotomy. Even with high MRI sensitivity, some patients with medial meniscus posterior root tears may be under- and over-diagnosed
Yim et al., 2013 [27]	Not reported	Not reported	Other factors affecting the outcomes of non-operative treatment, such as the patient’s occupation and lifestyle, were not assessed. Most participants were women
Noorduyn et al., 2020 [28]	Not reported	Cardiovascular events, neurological problems, internal medicine conditions, re-surgery on the affected knee, total knee replacement, and knee pain	Not blinded to treatment; determined based on IKDC and not based on PSFS; patients experiencing knee pain related to MRI-confirmed meniscus tears were recruited. The PSFS has not been validated in this population or in similar populations. PT protocol consisted of general progressive exercises for cardiovascular coordination, coordination, balance, and closed kinetic chain strength of the lower limb, rather than exercises focused on patient-selected relevant activities
Ikuta et al., 2020 [29]	Not reported	Not reported	Short-term results were studied over 6 months, and the medium- and long-term outcomes are yet unknown. Small sample size
Kudo et al., 2013 [30]	Not reported	Not reported	Participants applied to participate in the ET for knee OA and may have had a strong motivation to exercise. Exercise may be less effective in providing symptomatic relief in cases where flexion contracture is observed
Stensrud et al., 2015 [31]	Not reported	Not reported	There were no restrictions due to participation in leisure-time physical activities during the study period, and no differences were reported in terms of type, frequency, or intensity of leisure-time physical activities between the groups. Leisure-time physical activities were self-reported at follow-up, which is limited by recall bias and overestimating fitness level. There is a large difference in time between baseline and intervention initiation between the two groups. The LSI was not reported as an outcome measure in the current study despite common use to express both isokinetic muscle strength 48 and single-leg hop performance
Neogi et al., 2013 [32]	Not reported	Not reported	The average follow-up was not long enough. The effect of NSAIDs was not measured. Small sample size
Prati et al., 2017 [33]	Not reported	Not reported	The study lacked randomization; a small number of patients were treated. Only a 3-month follow-up was evaluated
Sonesson et al., 2020 [34]	Not reported	Not reported	The patients were not blinded to the treatment. Only 70% of patients completed the 5-year follow-up questionnaire
Katz et al., 2013 [35]	30% of patients assigned to the physical therapy group crossed over to the surgery group in the first 6 months	Mild or moderate severity adverse effects occurred in 13 participants in the physical-therapy group, including, death, pain from fall or other trauma, knee bursitis, knee pain, and pain in the back, hip, or foot)	Only 26% of eligible patients were enrolled. The study was not blinded
Başar et al., 2021 [36]	Not reported	Not reported	Did not perform long-term follow-up; small sample numbers; the relationship between treatment method and knee OA progression was not investigated

Additionally, both serious and non-serious adverse events following rehabilitation were reported. Serious adverse events included neurological problems, cardiovascular and other systemic conditions, reoperation on the affected knee, and TKR [13,24,28,35]. In contrast, non-serious adverse events that were reported included PT and exercise-induced falls, knee joint swelling, and lower extremity pain [13,24,28,35]. Importantly, limitations were reported in the studies included in this scoping review; many reported difficulty in blinding the examiner, a common limitation for rehabilitation interventions [13,28,35]. Additionally, several studies reported small sample sizes and short follow-up periods [33,36].

Medial Degenerative Meniscus Injuries

Rehabilitation for medial degenerative meniscus injuries was mainly programmed with neuromuscular and strength exercises (Table 3) [16,23,31]. The average duration of intervention by exercise was 12 weeks, with a relatively wide range of follow-up periods from three months to five years. Outcomes indicating the effects of exercise were pain and symptoms (KOOS subscale pain), physical function (muscle strength [isokinetic knee peak torque], OLH test, and 6-minute timed hop test), and ADL or QOL (KOOS subscale ADL) (Table 4) [16,23,31]. Intervention tended to improve short- and long-term pain; other KOOS symptoms such as swelling, restricted range of motion, and mechanical symptoms; physical function; and ADL or QOL. Specifically, symptoms such as swelling, restricted range of motion, and mechanical symptoms [37].

Medial Meniscus Posterior Root Tear

Rehabilitation using MMPRT was mainly programmed with neuromuscular, strength, and ROM exercises (Table 3) [25-27,29,32]. In addition to rehabilitation, medications administered included celecoxib and other NSAIDs, paracetamol, and tramadol [26,27,32]. Outcomes indicating the effects of exercise were pain and symptoms (VAS), physical function (Lysholm Knee Score), and ADL or QOL (IKDC score, KOOS subscales [sport/recreation, ADL and QOL], Tegner activity scale, and Lysholm activity scale). Some reports demonstrated significant short-term differences in intervention effects in pain and symptoms, physical function, and ADL or QOL, while others did not exhibit any differences. Specifically, Neogi et al. observed a significant difference of 0 mm (0-40, p = 0.03) in the final follow-up VAS test and 10 mm (0-70, p = 0.04) in the final activity follow-up [32]. In contrast, Ikuta et al. discovered that the VAS was -27.0 mm (-42.0 to -11.9, p = 0.004) for adapting alignment exercise, while muscle training and exercise had a symptom subscale KOOS of 15.7 points (6.4-24.9, p = 0.001); the results differed according to outcomes [29]. Additionally, Ahn et al. observed no significant short-term differences in the IKDC subjective score (44.73 ± 2.75-45.85 ± 14.00, p = 0.633) or the Tegner and Lysholm activity scales (51.62 ± 23.09-51.15 ± 22.67, p = 0.932) [25]. The limitations of these studies were small sample sizes and no medium- to long-term follow-up [25,29,32]. In contrast, a study that used combined rehabilitation and pharmacotherapy reported that it did not examine the effects of pharmacotherapy [32].

Cut-Off Scores and Clinically Important Differences

Three studies reported a clinically important difference (CID) in outcomes of meniscus injury and knee OA. Kise et al. reported a cut-off score for KOOS; Katz et al. described a CID for the WOMAC physical-function scale; and Noorduyn et al. reported minimal important change (MIC) for the PSFS [24,28,35]. Specifically, KOOS cut-off values were defined as 10.1 total points, 7.4 points for pain, 8.4 points for symptoms, 4.1 points for ADL, 10.9 points for sport/recreation, and 13.6 points for QOL [24]. Additionally, Katz et al. defined the CID of the WOMAC physical-function scale as 8 points [35]. Furthermore, Noorduyn et al. defined the MIC of PSFS as 2.5 points [28]. This review compared the data of the included studies using above the cut-off, CID, and MIC thresholds, which are listed in Table 5. Because the results of pain and symptoms, physical function, ADL, sport/recreation, and QOL are presented in this scoping review, the relevant items were summarized. In summary, two reports [16,30] evaluated the KOOS subscale for pain, one [16] evaluated symptoms, another [16] evaluated ADL, two [16,30] examined sport/recreation, and two [16,30] evaluated QOL. There was one report on CID based on the WOMAC physical function scale [35]. The MIC for PSFS was evaluated by one report [28].

Discussion

This scoping review summarized the effects of rehabilitation according to the degree of meniscus injury and described the existing gaps in the literature. We also summarized the effects of rehabilitation, focusing on pain, physical function, and ADL or QOL. We aimed to describe how these outcomes are influenced by rehabilitation in the short term, medium term, and long term. Studies that did not classify or distinguish the degree of meniscus injury have reported that the effects of rehabilitation were generally favorable in the medium-to-long-term duration. In contrast, for cases in which the intervention was not sufficiently effective, patients reportedly subsequently underwent APM or TKR. Additionally, studies on MMPRT have not provided a definite conclusion on the efficacy of rehabilitation due to the short intervention period. Conversely, cut-off values for assessment scores, CID, and MIC were reported only in studies that did not classify or differentiate the degree of meniscus injury. This review focused on these gaps in evidence and describes the potential areas that need to be addressed in the future.

Gaps in Research

Differences in interventions: There were differences in intervention procedures and methods among the exercises in the study that did not distinguish between the degree of meniscus injury and MMPRT exercises. Most of the exercise programs in the interventions that did not differentiate the degree of meniscus injury initiated with a warm-up involving a stationary bike and included exercises to improve physical function, focusing on strength, neuromuscular exercises, aerobic conditioning, functional mobility, and balance exercises [13,24,28,30,33-36]. Stationary biking was also programmed as a cool-down exercise at the end of each session [13,24,28]. In contrast, rehabilitation programs for MMPRT focused primarily on ROM exercises, muscle strength, endurance, and flexibility [25-27,29,32]. In addition to exercise, medication was also prescribed [26,27,32]. We consider this a gap because of the difference in methods between interventions that do not distinguish the degree of meniscus injury and MMPRT. Medications administered for MMPRT are considered an important intervention because they are recommended by the Osteoarthritis Research Society International guidelines [11]. However, to prove the effectiveness of rehabilitation alone, other interventions, such as pharmacotherapy, should be excluded. We believe that rehabilitation should be a stepwise program that improves physical function and reduces pain and symptoms [35,38,39].

Differences in outcomes: In studies that did not distinguish the extent of meniscus injury, pain/symptoms (EQ-VAS, KOOS subscales [pain and symptoms], and VAS or NRS), physical function (IKDC score, muscle strength, PSFS, ROM, and WOMAC physical-function score), and ADL or QOL (the EQ-5D, KOOS subscales [ADL, sport/recreation, and QOL], and SF-36 physical-activity scores) were utilized [13,24,28,30,33-36]. However, for MMPRT, the IKDC score, KOOS, and VAS, plus the Lysholm knee score and TAS score, were included as outcome measures. The Lysholm knee and TAS scores are used to evaluate outcomes of knee ligament surgery (e.g., anterior cruciate ligament, posterior cruciate ligament, medial collateral ligament, and lateral collateral ligament) and meniscal repair/meniscectomy [39-41]. Hence, we believe that the KOOS and WOMAC scores should be considered in lieu of the Lysholm knee or TAS scores as they can be used to determine the overall effects of the interventions on pain, physical function, ADL, and QOL. Although the overall intervention effects could not be evaluated in this scoping review, the cut-off, CID, and MIC were important clinical outcome measures in knee OA associated with meniscus injuries, which were discussed in five studies [16,29,33-35]. Additionally, apart from the cut-off, CID, and MIC, some studies on knee OA reported minimal CID (MCID) in the NRS, SF-36, VAS, and WOMAC scores [42]. Specifically, they reported an NRS of 1.0, an SF-36 (physical function) score of 3.3 points, a VAS of -8.4 to -19.9 mm, and a WOMAC (pain/physical function) score of -9.7/-9.3 points [42]. Moreover, minimal clinically important improvement (MCII) was reported as -19.9 mm for VAS and -9.1 (−26.0%) points for WOMAC physical function [43]. Conversely, some studies involving middle-aged and older patients with meniscus injuries have reported IKDC scores with MIC defined as 10.9 points [44]. Hence, we examined the studies reported in this review that met these definitions, and nine papers were included in this category [13,26,27,29,32-36]. The MCII is reportedly unaffected by age, duration of disease, or sex, and we believe that this is a useful definition that can be utilized in clinical practice [45]. Based on the above, we believe that the outcomes of knee OA associated with meniscus injuries should be evaluated by the KOOS subscales (e.g., pain/symptoms, physical function, ADL, sport/recreation, and QOL) or WOMAC scores, with the cut-off values, CID, MIC, MCID, or MCII applied in clinical practice.

Differences in follow-up periods: Medium- to long-term follow-up from one to five years demonstrated generally favorable changes over time [13,16,24,26-28,34,35]. In contrast, when the follow-up period was shorter (between three and six months), studies reported that outcomes differed depending on the rehabilitation modality [28]. Additionally, previous studies have reported that long-term non-operative therapy may, in some cases, delay total knee arthroplasty (TKA) [46,47]. Furthermore, it was reported that patients with knee OA who avoided surgery for five years after the onset of symptoms might have a worse prognosis than those who underwent TKA. Avoidance of surgery is not necessarily an indicator of the success of non-operative treatment in these patients [46]. In contrast, two-thirds of patients could delay TKR surgery for at least two years following non-surgical treatment for moderate-to-severe knee OA [48]. There is a difference in efficacy outcomes between short- and long-term follow-ups. When the follow-up was short-term, no consistent results were observed in the effects of the intervention. For long-term follow-ups, the response to intervention was generally positive, but a number of patients might transition to TKA/TKR. Furthermore, long-term non-operative management can delay TKA/TKR. Considering the above, when determining the length of follow-up, a comprehensive decision should be made based on the patient’s symptoms, needs, and outcome indicators to determine if TKA/TKR will be needed in the future.

Arthroscopic Partial Meniscectomy or Total Knee Replacement After Rehabilitation

There were three reports of conversion to APM or TKR after rehabilitation [13,24,35]. The reasons given included increased pain and decreased knee function and satisfaction [13,24]. In addition, patients with high WOMAC physical-function scale scores at six months post-intervention underwent APM or TKR treatment [35]. Therefore, any exacerbation of pain or decline in physical function after rehabilitation may have led to the transition to APM or TKR.

Clinically Important Differences

Cut-offs, CIDs, and MICs are important indices in knee OA, as they are associated with meniscus injury; these were examined in three studies [24,28,35]. In addition, a study on knee OA reported minimal CIDs (MCID) in NRS, SF-36, VAS, and WOMAC scores [42]. Specifically, they reported an NRS of 1.0, an SF-36 (physical function) score of 3.3 points, a VAS of -8.4 to -19.9 mm, and a WOMAC (pain/physical function) score of -9.7/-9.3 points [42]. Moreover, MCII was reported to be -9.9 mm for VAS and -9.1 (-26.0%) points for WOMAC physical function [43]. In contrast, some studies involving middle-aged and older patients with meniscus injuries reporting IKDC scores defined MIC as 10.9 points [44]. On the other hand, the MIC of the PSFS is reported at 2.5 points [28]. Hence, we identified the studies reported in this review that met these definitions; nine met these criteria [13,26,27,29,32-36]. The MCII is reportedly unaffected by age, duration of disease, or sex, and we believe that this is a useful definition for utilization in clinical practice [45]. Therefore, we believe that the outcomes of knee OA associated with meniscus injuries should be evaluated by KOOS subscales (including pain/symptoms, physical function, ADL, sport/recreation, and QOL) or WOMAC scores, and cut-off values, CIDs, MICs, MCIDs, and MCIIs should be applied in clinical practice.

Clinical Implications

Although this scoping review cannot distinguish and evaluate the degree of meniscus injuries, we believe that ET for knee OA associated with meniscus injuries should consist of a stepwise program of interventions from the acute to the sub-acute phases and finally to the advanced activity phase [35]. In addition, the cut-off values, CID, MIC, MCID, and MCII may have important applications in clinical settings.

Limitations

Our scoping review has some limitations regarding its methodology and interpretation of results. First, the inclusion criteria for this review incorporated medications in addition to rehabilitation; hence, it was impossible to examine the effects of rehabilitation alone. Studies examining MMPRT require cautious interpretation because they cannot present the effects of rehabilitation alone because pharmacotherapy is used for a certain period. Second, because most studies compared APM with rehabilitation, it is unclear whether there was a significant difference before and after the rehabilitation-only intervention. Third, the methods and effectiveness of rehabilitation at the patellofemoral joint (PFJ) are unknown; knee OA affects the PFJ and tibiofemoral joint OA. This review provides an important perspective and points for consideration given that meniscus tears reportedly increase the risk of PFJ OA [49]. Additionally, it is important to note that for tibiofemoral joint OA and PFJ OA, no specific interventional procedure is indicated.

Future research

Further intervention studies are needed regardless of the extent of the meniscus injury as the definition of the MCID is important to generalize the efficacy of rehabilitation alone in treating knee OA associated with meniscus injuries.

## Conclusions

Studies on rehabilitation of middle-aged and older patients with knee arthritis associated with meniscus injury had an evidence gap, with differences in intervention duration, methods, and outcomes. Additionally, at short-term follow-up, intervention effects varied across studies. Therefore, the rehabilitation approach should consist of a stepwise program that improves physical function and reduces pain and other symptoms. Furthermore, the follow-up period should be at least one year, which can be considered medium-to-long term, and outcomes should utilize the KOOS and WOMAC scores to improve pain, symptoms, and physical function, as well as ADL and QOL based on the cut-off scores, CID, MIC, MCID, or MCII definitions. Knee OA associated with meniscus injury may be a point of consideration in clinical practice and research as it may lead to a certain number of patients transitioning to surgical repair or replacement in the long term.
